# The Multifaceted Role of Mating Type of the Fungus and Sex of the Host in Studies of Fungal Infections in Humans

**DOI:** 10.3390/jof8050461

**Published:** 2022-04-29

**Authors:** Nada Kraševec

**Affiliations:** Department of Molecular Biology and Nanobiotechnology, National Institute of Chemistry, SI-1000 Ljubljana, Slovenia; nada.krasevec@ki.si

**Keywords:** antifungals, *Aspergillus* spp., *Cryprococcus* spp., *Candida* spp., fungal diseases, immune response, multidrug resistance, pathogenic fungi, inclusion of sex and gender in research

## Abstract

This review discusses the inclusion of sex and gender variables in studies of fungal infections in humans at the pathogen, host, and antifungal trial levels. The mating type of some fungi, or perhaps more likely the absence of the other, appears to be associated with some infections. Sexual and parasexual reproduction of some fungi is an important mechanism for the development of antifungal drug resistance. Host sex or gender influences the incidence of some infections such as aspergillosis, cryptococcosis, paracoccidioidomycosis, dermatophytosis, and candidiasis due to differences in immune response, behavior, and awareness for early detection and treatment. Participant sex (and age) is relevant not only in clinical antifungal trials but also in preclinical studies. The dimensions of sex and gender are important determinants throughout the fungal infection process and in approaches to prevent or treat these infections, as well as in development of antifungal drugs. Failure to consider sex and gender may be detrimental to the holistic understanding of the processes involved in fungal infection.

## 1. Introduction

Fungi have a tremendous impact on our daily lives, much more than most people realize. The fungal kingdom consists of 1.5 million species, while some estimates put the number of fungal species at 6 million, of which only about 5% have been described [1,2]. Fungi first appeared about 1.5 billion years ago and were among the earliest organisms domesticated by humans [3]. They serve as important biofactories for humans, although the vast possibilities offered by the compounds produced by fungi remain largely unexplored. Fungal biotechnology can facilitate the transition from a petroleum-based to a bio-based circular economy. Fungi can be used in the production of food, feed, chemicals, fuels, textiles, and materials for construction, transportation, furniture, and more in an efficient and sustainable manner [1,2].

However, fungi also produce toxins that can spoil food, and cause disease, especially in immunocompromised patients, but also in healthy people, driving up food safety and public health costs [1,2]. Only about 100 to 200 of all fungal species have been associated with human disease [3]. Invasion of the human body is a major challenge for fungal pathogens because they must be able to grow at high (human) body temperatures (i), reach target tissues by penetrating host tissue barriers (ii), digest and absorb components of human tissues (iii), and resist the human immune system (iv) [4]. Fungi that infect healthy humans devote a large part of their physiology to morphogenesis, the variability of cell shapes and the ability to switch between them, resisting or evading the immune system, and establishing a complex network of sensing and signaling systems to produce various proteins and compounds [5]. These proteins include digestive enzymes or lipid-binding pore-forming proteins or steroid-converting cytochromes P450 and other converting enzymes. During sexual development, various secondary metabolites are produced to protect the fungus from predators or hosts [6]. Understanding the pathophysiology of fungi in humans is particularly important for predicting the possible emergence of novel fungal pathogens that may result from global changes in the environment.

We are slowly becoming aware of the important role that gender plays not only in our daily lives, but also in scientific research. It was not so long ago that scientific research was conducted almost exclusively by men and for men, and the technical products developed were tailored to men or users, according to the traditional social roles of men and women [7]. Some funding agencies in Europe and in the United States, which seem to be one of the main drivers of research, have already recognized the importance of considering sex in basic and clinical research [8]. The aim of sex and gender analysis is to promote rigorous, repeatable, and accountable science. The inclusion of sex and gender variables in experimental design has already enabled advances in a number of disciplines [7]. There is a roadmap for sex and gender analysis across scientific disciplines available, and researchers, funding agencies, peer-reviewed journals, and universities are encouraged to coordinate their efforts to implement robust methods for sex and gender analysis [9].

Two different forums of researchers not originally involved in sex and gender research have recently discussed the dimensions of sex and gender in scientific research. These were the final conference of the Plotina project entitled “ReGendering science—For an inclusive research environment” [10] and the annual event of the Slovenian Commission for Equal Opportunities in Science entitled “The Overlooked Dimensions of Gender in Scientific Research” [7]. Researchers presented their experiences of integrating the gender dimension in their research and described the thought process, possible doubts, or surprises to explore to what extent the gender dimension is already considered in research today and where we still overlook it.

In this paper, the author would like to reflect on two different issues related to sex and gender. One is the inclusion of the mating type of fungal pathogens and the other is the sex and gender of the human host in studies of fungal infections. Fungi have very different modes of reproduction, which raises scientific interest in the reasons for this divergence, especially in comparison to other organisms and between different fungal species. Sex in fungi is a very interesting topic that has been covered in many (review) papers and is often chosen as an attractive topic for closing lectures at fungal conferences [3,11,12,13]. The author would like to highlight some viewpoints that might be overlooked in the overall picture, not in the sense of an exhaustive overview, but to provide some insight into where the inclusion of sex/gender might also be found. These include the relationship between fungal mating type and pathogenicity, the sex of human hosts, their immune response and awareness of fungal disease, the influence of sex (and age) of participants in antifungal trials, and the gender of researchers who uncover the mechanisms of fungal disease and publish on fungal infections. The latter aspect, the gender of the researcher, is addressed elsewhere (Kraševec, in preparation).

## 2. Sex of the Fungi That Cause Infections

Sex occurred early in evolution, and most eukaryotes enter the sexual cycle during their lifetime. Sex is costly and inefficient because nonidentical haploid gametes are required, meaning that two parent organisms are needed for each offspring. Maintaining two mating partners reduces the fitness of the parent organisms. However, the benefits of sex appear to outweigh the efficiency of mitotic reproduction. Most biologists believe that the purpose of sex is to create diversity among the offspring [3,11,12].

Mating types in fungi are the equivalent of sexes in humans. Just over a decade ago, pathogenic fungi were thought to reproduce only asexually, mitotically, and mostly clonally. However, accumulating genetic evidence in the genomes of fungal species indicates that most (human pathogenic) fungi have retained all the necessary predispositions for sexual reproduction (hidden sex). Fungi may have a heterothallic sexual cycle in which different mating types mate and promote outbreeding. Another possibility is a homothallic sexual cycle, in which organisms are self-fertile, promoting inbreeding. In fungal evolution, the transition between heterothallic and homothallic lifestyles is common. The transition from outcrossing to self-fertilization allows the species to expand in a niche where the probability of encountering the other mating type is low. Maintenance of mating partners is not required for parasexual reproduction. The advantages of mating similar cells are not obvious at first glance, but even a small amount of cross-fertilization is sufficient to maintain diversity. The heterothallic or homothallic sexual (including parasexual) process itself can directly influence virulence, the formation of resistant spores, and the modulation of host cell interactions [3,11,12,13].

In most cases, (more) infective strains have been identified at the species level when fungal pathogen has been identified; these strains have usually been isolated as asexual forms of the causative fungus. Current knowledge of the characteristics of sexual and asexual reproduction of fungal pathogens that more commonly infect humans in most regions studied, e.g., *Aspergillus*, *Cryprococcus*, and *Candida*, and of the diseases they cause, is briefly summarized here.

### 2.1. Aspergillus Diseases and Reproduction of the Fungus Aspergillus fumigatus

Fungi of the genus *Aspergillus*, e.g., *A. fumigatus*, are widespread, ubiquitous fungi that live both indoors and outdoors. While most people are exposed to the spores of the fungus *Aspergillus* on a daily basis without harm, immunocompromised individuals are at higher risk of developing health problems caused by this fungus. Since this fungus remains an important human pathogen, its biology, pathogenesis, molecular biology, and virulence factors of *A. fumigatus* have been described in detail [14,15,16,17,18,19,20]. The success of the spread of *A. fumigatus* has been attributed to the following factors: the ability to survive and grow under many environmental conditions (i), efficient aerial dispersal (ii), physical characteristics that allow conidia to reach distal sites of the respiratory tract (iii), and rapid adaptation to the host environment (iv) [11,21].

The disease caused by the fungus *Aspergillus* is referred to as aspergillosis, with some forms being mild and others severe [22]. Invasive aspergillosis causes severe infection of the lungs and can spread to other parts of the body; it usually affects people with a weakened immune system [22]. The number of cases of invasive aspergillosis caused by *A. fumigatus* is increasing due to the increasing number of medical procedures that compromise the ability of the patient’s immune system to control infections. These cases are difficult to diagnose, so treatment is often not initiated in a timely manner [23]. Chronic pulmonary aspergillosis is an infection of the pulmonary cavity that can be long-lasting [22]. Cutaneous aspergillosis occurs when the fungus enters the body through skin damage and causes infection, usually in people with weakened immune systems [22]. Allergic bronchopulmonary aspergillosis causes inflammation of the lungs and allergic symptoms such as cough and wheezing, while allergic aspergillus sinusitis causes inflammation of the paranasal sinuses and symptoms of sinusitis; neither leads to infection [22]. In addition, aspergilloma, a fungal nodule that grows in the lungs or sinuses, does not usually spread to other parts of the body

The filamentous fungus *A. fumigatus* reproduces mainly asexually, by mitotic division of haploid cells and by the formation of asexual conidiospores. In rare cases, heterothallic mating between MAT1-1 and MAT1-2 may also occur. During mating, cleistothecia are formed that contain multiple ascospores. In the fungus *A. fumigatus*, sexual reproduction and virulence are related: mating type MAT1-1 is associated with increased invasive growth and is more virulent than mating type MAT1-2. The formation of recombinant strains contributes to altered resistance to antifungal drugs [11].

Doubts have also been raised about the role of mating type in the virulence of *A. fumigatus*. Therefore, researchers generated nearly identical strains of *A. fumigatus* with opposite mating types, which allowed a more accurate test of whether different mating types have different virulence abilities. No differences in virulence were detected between these two strains of *A. fumigatus* in the two infected strains of mice and *Galleria mellonella* larvae, which may indicate that mating type does not affect virulence and the emergence of azole-resistant strains [23]. Infection of humans with these two recombinant strains for definitive confirmation is highly unethical.

### 2.2. Cryptococcal Diseases and Reproduction of the Fungus Cryptococcus neoformans

Cryptococcosis usually affects the lungs or central nervous system, but may affect other parts of the body. A brain infection caused by the Cryptococcus fungus is called cryptococcal meningitis. Most people infected with the fungus *C. neoformans*, which is common worldwide, usually have a weakened immune system, especially those with advanced HIV/AIDS [22].

The cells of *C. neoformans* are haploid and can divide asexually or enter a heterothallic or homothallic reproductive cycle [11]. In heterothallic mating, pheromone signals between cells a and α lead to cell fusion, but the nuclei do not fuse; instead, filamentous, binucleate hyphae are formed. The tips of the filamentous cells develop into bases, in which nuclear fusion and meiosis then occur. Further mitotic division cycles give rise to multiple haploid basidiospores that form four long chains [11]. The effects of sexual reproduction on the virulence of the fungus *C. neoformans* are diverse. Most clinical isolates are of the MATα type, so a difference in virulence of the mating types is suspected. Both mating types can cause disease, but MATα is more efficient than MATa [11]. Pheromone signaling causes growth in the form of hyphae and the formation of titanium cells. Basidiospores are formed, which are the infection pathway (in addition to encapsulated yeast-like cells). Hybrid and recombinant strains can contribute to antifungal resistance and fitness in some cases [11].

### 2.3. Candida Infection and Reproduction of Candida albicans

*Candida* normally lives on the skin and in the body, such as the mouth, throat, intestines, and vagina, without causing problems; however, occasionally it can cause infections. Candidiasis of the stomach or oropharynx develops in the mouth or throat. A yeast infection is candidiasis in the vagina. Invasive candidiasis occurs when *Candida* species enter the bloodstream or infect internal organs such as the kidneys, heart, or brain [22].

The cells of *C. albicans* are diploid and can divide asexually or mate hetero- or homothallically [11]. The mating cells of MTLa and MTLα must switch from the white phase to the opaque phase to become compatible. The opaque cells secrete pheromones that cause the formation of conjugation tubes, which lead to cell and nuclear fusion and form tetraploid cells. Homothallic mating may also occur. The mating products usually result in intentional loss of chromosomes to return to the diploid state. The MTLa/α mating type is more virulent than MTLa/a or MTLα/α [11]. The transition from the white to the opaque phase affects niche selection, interaction with immune cells, filamentous growth, and pheromone-induced biofilm formation. Under certain conditions, the formation of recombinant strains contributes to antifungal drug resistance and fitness [11].

Sexual reproduction and associated processes play a somewhat limited but important role in generating genetic diversity in *Candida* species and other fungal pathogens. These processes also contribute significantly to the evolution of pathogenicity and drug resistance.

The most recently emerging pathogen species, *C. auris*, has caused several large outbreaks of healthcare-associated infections worldwide and was first described in 2009 [24]. The fungus *C. auris* is resistant to several antifungal drugs and has become a major cause of invasive candidiasis in some hospitals. It differs from most other pathogenic *Candida* species in its resistance to antifungal drugs and its ability to spread among patients. More than most *Candida* species, *C. auris* tolerates an extremely saline environment and higher temperatures. Factors that could lead to its spread, including the possible role of public health, antifungal drug use, and environmental changes, including human activities that could increase the occurrence of *C. auris* in the environment or allow increased contact with humans, are currently under investigation [25].

What is the evidence for sexuality in *C. auris*? *Candida auris* and its closest relatives should have sufficient mating and meiosis factors to support the sexual cycle. Indeed, the mating locus in *C. auris* is complete and both mating types are present, suggesting that *C. auris* is capable of mating and meiosis, or at least mating and coordinated chromosome loss (parasex). It cannot be ruled out that *C. auris*, like *C. albicans*, can reproduce unisexually with isolates of the same strain. To confirm that *C. auris* can reproduce sexually, it is extremely important to determine the natural environment, as it could be further delineated if different mating types exist in the natural population [26].

The fungus *C. auris* is genetically most closely related to the rarely observed and often resistant species *C. haemulonii*. Nearly complete genomes of four clades of *C. auris* have been identified, including rearrangements of seven chromosomes within clades and between species. Most mating and meiotic genes are conserved, and strains contain separate mating loci of MTL or MTLα. Due to karyotype differences among isolated *C. auris* clades, existing clinical strains of *C. auris* are unlikely to mate successfully. However, these differences do not rule out parasexual mechanisms. Based on current data, they suggest that health risk is not increased by *C. auris* mating and resulting diversity in the clinical context [27].

## 3. Difference of Sex and Gender of the Human Host in Susceptibility to Fungal Infection

Depending on biological characteristics, organisms can generally be classified as female, male, intersex, and hermaphrodite. Biological sex describes sexual distinction that goes beyond mere reproductive function and includes appearance, physiology, or neuroendocrine, behavioral, and metabolic systems [9]. Sex differences in humans are the result of a complex interplay of sex hormones, genetic variability, and the environment against a background of intrinsic effects of sex chromosome differences. Indeed, each adult human somatic cell exhibits sex-specific differences in gene expression and epigenetic profile to varying degrees. The long-recognized differences between men and women in health, longevity, disease risk and progression, and response to therapies have a genetic and epigenetic basis [28].

Gender refers to psychological, social, and cultural factors that shape attitudes, behaviors, stereotypes, technologies, and knowledge. It refers to spoken and unspoken rules regarding gender in society and how people in different cultures perceive themselves [9]. It is important to note that sex and gender are related in various unexpected ways. For example, perception of pain shows biological differences in signaling physiology, but also includes sociocultural components of how women, men, or people of different genders report pain, and how physicians understand and treat pain depending on the patient’s perception of gender [29]. Pain perception is an important factor in early detection and timely initiation of treatment for fungal infections.

The Gender and Gender Equality in Research guidelines (SAGER) emphasize the correct distinction between sex and gender to avoid confusion [30].

### 3.1. Is Awareness of Invasive Fungal Diseases Independent of Gender?

Because invasive fungal diseases cause significant morbidity and mortality, awareness is critical for early diagnosis and treatment. Public awareness of invasive fungal diseases was low in the 2019 U.S. study, with approximately two-thirds of respondents having never heard of any of the fungal diseases included in the study [31]. The least known fungal disease was blastomycosis (4.1% of participants), followed by aspergillosis, histoplasmosis, coccidioidomycosis, and cryptococcosis, and the best known was candidiasis (24.6%) [31]. Individuals who knew one fungal disease were more likely to recognize the others. Male sex, higher educational level, and higher number of prescribed medications were associated with higher knowledge of fungal diseases overall. Women were more than three times as likely as men to recognize candidiasis [31]. Further educational efforts are needed to increase people’s awareness of fungal infections.

### 3.2. Who Can Get a Fungal Infection?

Fungi are common in the environment, and every day people breathe spores in or are exposed to fungi without getting sick. Anyone can get a fungal infection, even otherwise healthy people, but many of the fungal infections are classified as opportunistic. Some people are born with a weakened immune system; others suffer from a disease that impairs their immune function. In addition, some medications can interfere with the ability to fight infections [22]. Most fungal infections occur in people who are already seriously ill, and often jeopardize even the success of recent medical advances in cancer treatment, organ and hematopoietic stem cell transplantation, neonatal medicine, autoimmune disease treatment, trauma and critical care, and sophisticated surgery [4].

Although *Candida* and *Aspergillus* species remain the most common causative agents of invasive fungal diseases, there has been a worrying increase in regional fungal diseases and infections with invasive fungi of the genera *Blastomyces*, *Coccidioides*, *Histoplasma*, *Cryptococcus*, *Pneumocystis*, and *Sporothrix* [32]. New fungal diseases caused by fungi from different taxonomic groups are constantly emerging. The human fungal pathogens affecting healthy or immunocompromised individuals have been identified in several taxonomic groups: phylum Ascomycota, e.g., Chaetothyriales, Eurotiales, Hypocreales, Microascales, Mycosphaerellales, Onygenales, Ophiostomatales, Pleosporales, Pneumocystidales, Saccharomycetales, and Venturiales; phylum Basidiomycota, e.g., Malasseziales, Tremellales, and Trichosporonales; subphylum Entomophthoromycota, e.g., Entomophthorales; and phylum Mucoromycota, e.g., Mucorales [4,33,34]. Not much is known about the relationship between mating type and infection in some of these species.

### 3.3. Is There a Sex-Dependent Difference in the Human Immune Response?

The sex of the human host influences the immune response to various antigens, e.g., fungi, viruses, bacteria, parasites, and allergens, and shows differences in innate and adaptive immune responses. Differences in immune response can be influenced by both sex and gender, with sex contributing to physiological and anatomical differences that affect fungal exposure, recognition, clearance, or transmission [35]. Some immunological differences between sexes persist throughout life, while others become apparent only after puberty and before reproductive aging, suggesting the involvement of genes and hormones. Conversely, gender may reflect behaviors that affect fungal exposure, access to healthcare, or mode of seeking medical help, which in turn influences the course of infection. In addition, early microbiome exposure also affects infections [35]. Female cyclic immunity should be considered to understand the phenotypic diversity of female behavioral immunity and reproductive behavior, male immunity, and the evolution of sex-specific pathogen virulence [36]. The resulting immunological differences contribute not only to differences in susceptibility to infectious diseases caused by fungi and other pathogens and response to vaccines in men and women, but also to the greater incidence of cancers or autoimmune diseases associated with fungal infections than in healthy individuals.

In general, sex and age influence: susceptibility to infection (i), modulation of immune response (ii), immunosenescence (iii), and response to vaccination (iv) [37]. For many infectious diseases, the rate of infection or mortality is higher in men than in women, with some exceptions, such as sexually transmitted diseases. Some infectious diseases are equally prevalent but more severe in women, e.g., measles, toxoplasmosis, dengue, or hantavirus. Aging alters the sex difference in part through the contribution of hormones (i) [37]. Estrogens significantly strengthen the immune system. Androgens and progesterone have mainly immunosuppressive effects. The effects of sex steroid hormones are observed on both adaptive cells, e.g., CD4 + and B cells, and innate cells, e.g., natural killer cells, macrophages, and dendritic cells. Sex hormones affect cytokine secretion and the balance of cytokine profiles of T helper lymphocytes. Estrogens also increase the production of high-affinity immunoglobulins (ii) [37]. The immune and endocrine systems change with age, but the aging of the immune system in women and men is different. Menopause has a particularly strong effect on the immune system in women. Hormone replacement therapy partially reverses the effects of aging on the immune system and returns it to premenopausal levels, confirming the effect of hormones (iii) [37]. The immune response to some vaccines differs between women and men. Women often have a stronger humoral response, for example, to influenza and hepatitis B. Men may also have a stronger response, e.g., to pneumococcal polysaccharide vaccines. With age, the sex difference changes to some degree, suggesting a contribution from hormones. Animal models suggest that hormone replacement therapy may reverse vaccine efficacy in premenopausal levels (iv) [37].

The sexes differ in the intensity (i.e., pathogen load), prevalence (i.e., proportion of the population with disease), incidence (i.e., new cases), and severity (i.e., hospitalization or progression) of diseases caused by fungi, viruses, bacteria, and parasites. Men are generally more susceptible to these infections than women, but the reasons for the higher susceptibility in men are diverse [38,39]. Five types of fungal infections are considered here: aspergillosis, cryptococcosis, paracoccidioidomycosis, dermatophytosis, and candidiasis.

### 3.4. Susceptibility to Infection with the Fungus Aspergillus fumigatus by Sex/Gender

Sex differences in the anatomy and physiology of the respiratory system have been widely reported. These intrinsic sex differences have also been shown to influence the pathophysiology, incidence, morbidity, and mortality of various pulmonary diseases across the lifespan [40].

One study examined the incidence and development of invasive pulmonary aspergillosis and galactomannan testing in patients with aspergillosis infections [41]. For the incidence of invasive pulmonary aspergillosis, a male-to-female ratio of 1.85:1.15 was obtained. An increasing trend of invasive pulmonary aspergillosis was observed over time in both men and women. Galactomannan testing is recommended for early diagnosis of patients with suspected aspergillosis. The increase in the incidence of invasive pulmonary aspergillosis may be positively related to the increase in galactomannan testing over the past decade, with these tests being performed more frequently in men than in women [41].

In invasive pulmonary aspergillosis of patients with viral SARS-CoV-2 infection, the possibility of colonization is the most important confounder rather than invasive disease [42]. The vast majority of patients did not have any of the classic host risk factors, such as immunosuppression due to organ transplantation or neutropenia, although a significant proportion (half) had received corticosteroids. Male sex, age, and pulmonary comorbidities were associated with higher mortality. Mortality was generally lower in patients treated with voriconazole. In critically ill patients with coronavirus disease 2019 (COVID-19) who do not improve, clinical surveillance for associated pulmonary aspergillosis is advisable, even in patients who do not meet classic host criteria for invasive mycoses, especially if they are receiving corticosteroids [42].

Another study aimed to determine the frequency of invasive pulmonary aspergillosis in patients with COVID-19 admitted to the intensive care unit, to describe the characteristics of patients with invasive pulmonary aspergillosis, and to evaluate its impact on prognosis [43]. Invasive pulmonary aspergillosis is a relatively common complication in severe patients with COVID-19 and is responsible for increased mortality. Probable invasive pulmonary aspergillosis was diagnosed in 5.7% of patients with COVID-19 admitted to the intensive care unit and in 19.4% who had a respiratory sample taken due to an exacerbation. No significant differences were observed between patients with and without invasive pulmonary aspergillosis in terms of age, sex, medical history, and severity on admission and during hospitalization. Azithromycin, which is known to have immunomodulatory properties, may contribute to increasing the susceptibility of patients with COVID-19 to invasive pulmonary aspergillosis. The propensity for invasive pulmonary aspergillosis and its occurrence were observed at high doses of dexamethasone. All-cause mortality was higher in patients with invasive pulmonary aspergillosis [43].

Given the increasing incidence and mortality of influenza-related aspergillosis, a study summarized risk factors, clinical features, and prognostic factors for the development of aspergillosis in immunocompetent influenza hosts to further investigate the high-risk population and improve outcomes [44]. The study showed that coinfection with aspergillosis increased all-cause mortality in severe influenza from one-quarter to one-half of cases, along with higher white blood cell, neutrophil granulocyte, procalcitonin, and lower CD4 + T-cell counts in the death group. Sex, age, underlying disease, use of immunosuppressant and steroids, and CD4+ T-cell count did not affect the incidence of influenza-associated aspergillosis. However, cases of influenza-associated aspergillosis are usually more likely to have H1N1 subtypes and higher levels of C-reactive protein and interleukin-6 than cases without aspergillosis. Concurrent aspergillosis infection in patients with severe influenza can lead to markedly increased mortality associated with severe respiratory failure due to mixed infection and immunosuppression. Excessive inflammatory response in the lungs has been associated with coinfection with invasive pulmonary aspergillosis [44].

Male sex, advanced age, low body mass index, chronic obstructive pulmonary disease, systemic steroids, *Mycobacterium* abscesses complex as the etiologic organism, and the fibrocavitary form of nontuberculous mycobacterial lung disease remained significant predictors for the development of chronic pulmonary aspergillosis in patients with nontuberculous mycobacterial lung disease [45].

Allergic bronchopulmonary aspergillosis commonly affects patients with cystic fibrosis and asthma; it often occurs at a young age and is not sex-specific [46]. *Aspergillus fumigatus* frequently colonizes the airways of patients with cystic fibrosis and can cause severe disease such as allergic bronchopulmonary aspergillosis, *A. fumigatus* bronchitis, or even *A. fumigatus* pneumonia. Urban living should be considered as a possible new risk factor for *A. fumigatus* colonization of the airways of cystic fibrosis patients [47].

Clinically, gender differences were observed in children with asthma and allergic diseases, with a higher prevalence of asthma in males before puberty [48]. Blood was collected each year, and mononuclear cells were stimulated with phytohemagglutinin. The concentrations of interferon-β, interleukin-5, -10, and -13 in the supernatants were determined by immunoassay. Total and allergen-specific IgE were measured. There are gender differences in the expression of the atopic phenotype and in vitro immune responses between boys and girls in the prepubertal school years. Boys aged six to nine years have higher rates of atopy and altered cytokine responses to phytohemagglutinin stimulation compared to girls [48].

### 3.5. Susceptibility to Infection with the Fungus Cryprococcus neoformans by Sex/Gender

Cryptococcosis affects a quarter of a million people each year and results in more than 180,000 deaths [49]. Men are more frequently affected by cryptococcosis than women, a phenomenon observed more than half a century ago. The gender imbalance is also observed in the non-HIV-infected population, where the ratio is about three males to one female, compared to the HIV-positive population, where the ratio is about four males to one female [38,49,50]. Male sex is considered a risk factor for cryptococcosis. Men who have the disease have more severe symptoms and poorer treatment outcomes. There are a number of observational, clinical, and epidemiologic studies documenting greater male involvement in *C. neoformans* infections, but there is no further explanation of the cause or mechanism. The very limited primary research suggests that sex hormones are likely the cause. Given that sex differences are widespread and accepted by many researchers, it is surprising that this is not more widely known [49].

Resistance to the pathogenic fungus *Cryptococcus neoformans* is sex-dependent. The nematode *Caenorhabditis elegans* consists of a population of self-fertilizing hermaphrodites with occasional males that differ anatomically and behaviorally from hermaphrodites. They also differ in their susceptibility to the fungal pathogen *C. neoformans*. Wild-type males exhibit greater resistance to this pathogen than do hermaphrodites, and this resistance may be caused by insufficient activation of the male sex determination pathway in hermaphrodites. Resistance is determined at the molecular level by overlapping pathways that control immunity and longevity, and is not due to behavioral changes or reproductive differences [51].

### 3.6. Susceptibility to Infection with the Fungus Paracoccidioides brasiliensis Is Sex/Gender Dependent

Paracoccidioidomycosis is a tropical lung disease caused by the dimorphic fungus *Paracoccidioides brasiliensis* and is the most common invasive mycosis in Latin America. Pleomorphic disease can be broadly divided into two forms, acute/subacute and chronic, and an asymptomatic form. The diversity of clinical manifestations is attributed to the increased pathogenicity of some strains of *P. brasiliensis* and, more importantly, to host factors that modulate the immune response to the fungus. The incidence is thought to be similar in both sexes and may be related to agricultural work leading to higher exposure to the fungus in the soil [52].

Most patients (three quarters) had the chronic form (adult type) of paracoccidioidomycosis, which presented as chronic lung disease, oropharyngeal and/or upper respiratory tract ulcers with or without regional lymph node enlargement. The chronic form of paracoccidioidomycosis is known to be more common in males, and the clinical disease occurs in adults in an extremely high male-to-female ratio of more than (11–15):1 [53,54,55].

The acute/subacute form (juvenile type) of the disease accounts for a quarter of cases. It is a common disease characterized mainly by generalized nodular enlargement with or without hepatosplenomegaly, skin, bowel, or bone lesions. It is directly related to female sex and inversely related to age. Although females are less likely to develop paracoccidioidomycosis after puberty, they are more susceptible to the acute/subacute form after acquiring the disease. This corresponds to a male-to-female ratio of (2–3):1 [52]. Paracoccidioidomycosis animal models show the same distribution of invasive pulmonary mycosis, however, sex differences in the immune response in humans remain to be studied in detail [54,56,57].

### 3.7. Incidence of Dermatophytosis Associated with Differences in Lifestyle between Women and Men

Tinea, or dermatophytosis, is a common infection of the skin and nails caused by about 40 different species of fungi from genera such as *Microsporum*, *Trichophyton*, and *Epidermophyton*. The infection can cause an itchy, red, circular rash; the different forms of tinea are usually named after the location of the infection on the body [22].

Onychomycosis (tinea unguium) can occur in both sexes, but most studies have shown that onychomycosis is more common in men [58]. Nail lesions and thickened nails are more common in men than in women. Women seem to be more conscious about their nails; however, because they wear tight shoes more often than men, deformity of the fifth nail is more common in women. In addition, due to oxidation caused by the use of nail polish products that darken the nails, treatment in women appears less favorable than it actually is. Although there are insufficient data on the actual effect of nail polish use on drug penetration, some physicians advise their patients not to use nail polish during topical treatment. One study showed better results with the use of the topical antifungal drug efinaconazole in women [59]. The incidence of tinea pedis increases with age and is higher in men than in women [58]. Men are affected about three times more frequently than women. This is probably due to different habits of wearing shoes (sports shoes or open shoes), foot hygiene, and occupational differences between the genders [58]. The fungus *T. rubrum* is the most common cause of tinea cruris, which is almost exclusively a male disease due to the moist environment created by contact between the scrotum and the skin of the groin [58]. Tinea cruris has also been found in female sex workers [58]. Tinea capitis: there is no difference in infection with the fungus *T. tonsurans*, as men are affected in a similar manner to women [58]. When infected with the fungus *M. canis*, males are more frequently affected than females [58]. If tinea capitis is caused by the fungus *T. schoenleinii*, it can occur in children and adults and affect males and females equally [58]. Adults who have had close contact with infected children are more likely to transmit the disease; shorter scalp hairs are more contagious, so the carrier stage and infection are more likely to be transmitted in men than in women. The prevalence of tinea manuum is less common and occurs slightly more frequently in men than in women [58]. This gender difference can be explained by occupational differences. Tinea manuum is observed in occupations where the palms are used intensively, and these occupations are common in men [58].

### 3.8. Incidence of Candidiasis in Relation to Life Circumstances

Factors that favor colonization with *C. albicans* include various life circumstances (advanced age, high-carbohydrate diet, newborns, pregnancy, smoking, stress, and urban lifestyle), diseases (AIDS, *Clostridium difficile* infection, dental caries, dentures, inflammatory bowel disease, and primary sclerosing cholangitis), and xenobiotics (antibiotics, cancer treatment, H2 receptor blockers, immunosuppression, proton pump inhibitors, and oral contraception). After menopause, the likelihood of *C. albicans* colonization decreases [60]. Diabetes mellitus, immunosuppression, malignancy, pregnancy, renal insufficiency, and xerostomia are factors that promote oral candidiasis [58]. Some studies have shown that women with denture stomatitis, another form of chronic oral candidiasis, are more commonly affected than men [58]. Candida folliculitis is an infection of the hair follicles of the beard and mustache in men [58]. Frequent contact of hands with water is associated with paronychia and candida onychomycosis, which is more common in women [58]. Fungal infections of the mucous membranes associated with an inflammatory host response are very common and can severely affect the quality of life for many people. The second most common cause of vaginitis is candida vulvovaginitis, which affects three quarters of women of childbearing age at least once in their lifetime, while almost one in ten of them suffers from a recurrent event [58,61]. Risk factors for candida vulvovaginitis are associated with elevated estrogen levels due to oral contraceptive use and pregnancy [58,60,62]. The vaginal bacterial microbiota also correlates with the menstrual cycle and sexual activity [63]. Candida balanitis is thought to be acquired through sexual contact with a partner who has candida vulvovaginitis [58]. Uncircumcised men have a higher incidence of balanitis. The risk factor for recurrent vulvovaginal candidiasis or onychomycosis may also be in genetics; the poorly expressed early-stop codon mutant in the β-glucan receptor dectin-1 did not mediate β-glucan binding and resulted in defective production of cytokines (interleukin-17, tumor necrosis factor, and interleukin-6) after stimulation with β-glucan or *C. albicans* [64].

The fungus *C. albicans* contains an estrogen-binding protein with a high affinity for estradiol, which may act as a potential receptor for estrogen and promote a number of different processes [20]. Estrogen promotes the morphological transition to a hyphal form, which may increase the virulence of the fungus via NADPH oxidoreductase- or heat shock protein-90-related pathways. Estrogen enhances fungal drug resistance by upregulating the expression of *C. albicans* drug resistance genes or the transcription of phosphatidylinositol transfer protein 16 [65]. Estrogen also affects the intravaginally infected female host: On the one hand, estrogen affects the vaginal epithelium by causing increased glycogen production, epithelial remodeling, and increased adhesiveness (focal adhesion kinase is involved). On the other hand, estrogen affects the transepithelial migration of neutrophils (CD44, CD47, and Cxcl1 are involved) and the ability to kill neutrophils (heparan sulfate is involved). As a result, *C. albicans* can survive and attach to the vaginal mucosa much more easily [65,66].

## 4. The Impact of the Sex of Participants in an Antifungal Trial

Trial results obtained in single-sex studies are sometimes extrapolated to both sexes without thorough justification. This can result in large economic losses and unintended deaths. Between 1997 and 2000, the U.S. Food and Drug Administration removed 10 prescription drugs with serious adverse effects from the market. Eight of the ten drugs were associated with significant health risks for women, and the primary reason for this problem was a male bias in basic, preclinical, and clinical studies. To achieve accurate and reproducible results that apply to both men and women, sex should be considered as an important biological variable in basic and preclinical research. Adverse effects of these drugs on women only became known through post-marketing reports [30]. Sex influences cause of death and morbidity. Sex differences are found in diagnosis and treatment, epidemiology, pathophysiology, clinical manifestations, disease progression, and response to treatment. Genetic, epigenetic, and hormonal influences of biological sex affect physiology and disease; on the other hand, gender affects community, physician, and patient behavior in the healthcare system [67].

### How Can Sex Differences Be More Clearly Highlighted in Cell-Based Studies and Later Preclinical and Clinical Studies?

Because different mechanisms between the sexes clearly have a profound impact on susceptibility, severity, and response to disease, the challenges of accounting for both sexes in infectious disease research must be addressed. The simplest step researchers can take is to specify the sex of the animals, cells, or cell culture models used [68]. Including sex as an important biological variable is essential to improve the accuracy and reproducibility of cell-based studies that provide fundamental information for subsequent preclinical and clinical trials. Funding agencies such as the European Commission, the Canadian Institutes of Health Research, and the U. S. National Institutes of Health seek to encourage researchers to consider sex not only in clinical research but also in basic and preclinical research [30]. The guidance SAGER emphasizes the need to distinguish between subjects by sex, to analyze results by sex, and to identify meaningful differences whenever possible, even if they were not initially expected [30]. The guidelines also include a series of questions to help authors prepare and review their work to determine whether they have adequately addressed sex/gender issues [69].

In one of the journals, the authors of the paper examined cell sex disclosure in relevant articles published in 2018 in the same journal to compare the progress of cell sex disclosure in articles from the original 2013 analysis. The results show that cell sex disclosure has improved from a quarter of publications to half of publications in 2018 [70]. However, sex disclosure is still frequently omitted or there is a bias in favor of the male sex. Omission of sex is more common in cell lines than in primary cells. In addition, the results obtained are rarely analyzed by sex, even when both male and female cells were used in the experiments [70]. Suppliers in the cell market also ignore the sex of the cells: about one-sixth of human cell lines sold by three renowned cell suppliers (American Type Culture Collection, European Cell Culture Collection, and Japanese Research Biological Resources Collection) since 2014 are without sex determination. Animal cell lines lack sex determination more frequently than human cell lines and most primary and stem cells [71].

## 5. Antifungals and Fungicides and Known Resistance Mechanisms

Four classes of antifungal agents are used to control fungal infections in humans (and animals): azoles, echinocandins, pyrimidine analogs, and polyenes. Azoles, which we also use as fungicides, interfere with the biosynthetic pathway of ergosterol. Echinocandins inhibit cell wall biosynthesis, pyrimidine analogs interfere with nucleic acid biosynthesis, and polyenes bind ergosterol [72]. In addition to azoles, the five major classes of plant protection fungicides include: morpholines, benzimidazoles, strobilurins, and succinate dehydrogenase inhibitors [72]. Morpholines inhibit two targets in the ergorsteol biosynthetic pathway, Δ14-reductase and Δ8-Δ7-isomerase. Benzimidazoles disrupt the cytoskeleton by binding to β-tubulin and prevent microtubule formation. Strobilurins (QoI) and succinate dehydrogenase inhibitors (SDHI) inhibit the electron transfer chain of mitochondrial respiration, with the SDHI inhibitory complex II (succinate dehydrogenase) and the QoI inhibitory complex III. Anilinopyrimidines interfere with mitochondrial signaling pathways [72].

Several resistance mechanisms to available antifungal agents have evolved in fungal pathogens. The resistance mechanisms that occur in fungal pathogens include: conformational changes in the target (i), increased expression of the target (ii), absence of the target (iii), increased pump exhaustion (iv), regulation of the stress response (v), and plasticity of the genome—aneuploidy or hypermutation (vi) and unknown mechanisms (vii) [72]. Of particular concern is the resistance of the pathogens *A. fumigatus*, *Candida albicans*, *C. auris*, *C. glabrata*, *Cryptococcus gattii*, and *C. neoformans* to azole agents. The maps of the occurrence of resistance of crop pathogens to azoles largely overlap with the maps of the occurrence of resistance of human and animal pathogens [72]. Three examples of antifungal activity, namely posaconazole, fluconazole, and emerging retinoids, are discussed below.

### 5.1. Posaconazole Is Well Tolerated Regardless of Age, Sex, Race, or Ethnicity

Posaconazole is a triazole antifungal agent used to treat invasive fungal infections and oropharyngeal candidiasis [73]. Two studies in healthy adults (over 18 years of age) investigated the effects of sex and age on the steady-state pharmacokinetics of posaconazole. Sex and age had no clinically relevant effects on the pharmacokinetics of posaconazole. Posaconazole is safe and well tolerated. In healthy adults, different dosing according to sex and age is not required [73].

### 5.2. Fluconazole Stimulates Fungi to Reproduce Sexually

Fungal infections are also commonly treated with fluconazole, which inhibits ergosterol synthesis in *Candida* [74]. Ergosterol corresponds to cholesterol in humans. The main mechanisms of fluconazole resistance have already been studied. The fungus can become resistant to fluconazole by a number of mechanisms. Highly resistant strains of *C. albicans* that cannot be treated with fluconazole usually use a combination of resistance mechanisms. Normally, *C. albicans* reproduces asexually by cell division. In the presence of fluconazole, the fungal cells can rapidly switch to sexual reproduction. In the offspring, the different resistance mechanisms are recombined, making the fungal population even less sensitive to fluconazole. Fluconazole not only allows selection of mutations leading to resistance, but can also lead to changes in the genome that make the normally asexual fungus “mating competent” [74].

Comparison of isolated genomes of *C. auris* and other *Candida* species associated with global public health outbreaks identified genes associated with drug resistance and virulence, including extended families of transporters and lipases, or mutations and copy number differences in ERG11. Gene expression analysis identifies transporters and metabolic regulators specific to *C. auris*, as well as those in related species that may contribute to the specific response to antifungal drugs [27].

### 5.3. Retinoids in Fungal Infections

Recently, however, it has been discovered that all-trans-retinoic acid has a direct fungistatic effect on fungi such as *C. albicans* and *A. fumigatus* [75]. All-trans-retinoic acid is an active metabolite of vitamin A with anti-inflammatory and immunoregulatory properties due to its ability to stimulate both innate and adaptive immunity, as well as its effects on proliferation, differentiation, and apoptosis in a variety of immune cells. The discovery of a direct fungistatic activity combined with the reported immunomodulatory properties makes all-trans retinoic acid an excellent candidate for new combined antifungal strategies for the treatment of systemic mycoses in immunocompromised and cancer patients [75]. In addition, determination of serum levels of all-trans-retinoic acid or vitamin A should be considered as a predictive marker for the development of fungal infections in psoriasis patients treated with interleukin-17 inhibitors [76]. Retinoids, either alone or in combination with currently available antifungal drugs, may be promising preventive or therapeutic antifungal agents to overcome the troubling drug resistance of pathogenic fungi [77].

## 6. Conclusions

A particular mating type of some fungal pathogen species appears to be more frequently associated with some human infections; direct studies in humans to confirm this association are not ethically defensible. Parasexual and sexual reproduction of some fungi is an important mechanism for the development of resistance to antifungal drugs. Some antifungal drugs induce fungi to reproduce sexually. The sex of human hosts affects the incidence of some fungal infections because the immune response is sex-dependent; this needs further investigation in studies. Awareness of early detection and treatment of fungal infections also depends on gender; the general population needs to be better educated about fungal infections. Sex and age of participants in antifungal trials are critical for interpretation of results, but researchers should report the sex of model test cells (primary cells) or cell culture models and model animals as early as preclinical studies; some funding agencies already require researchers to do so. The editorial policies of some journals, including this journal, follow the guidelines of SAGER, which require that subjects be disaggregated by sex, that results be analyzed by sex, and that meaningful differences be identified whenever possible, even if they were not initially expected; other journals should follow these guidelines as well. Globalization and climate change will exacerbate the challenges that fungal infections in humans already pose; we need more multidisciplinary approaches to address them.
